# A Nuclear Ribosomal DNA Phylogeny of *Acer* Inferred with Maximum Likelihood, Splits Graphs, and Motif Analysis of 606 Sequences

**Published:** 2007-02-17

**Authors:** Guido W. Grimm, Susanne S. Renner, Alexandros Stamatakis, Vera Hemleben

**Affiliations:** 1 Institute of Geosciences, Department of Biogeology and Applied Paleontology, University of Tübingen, Germany; 2 Department of Biology, University of Munich, Germany; 3 Swiss Federal Institute of Technology, School of Computer & Communication Sciences, Lausanne, Switzerland; 4 Center of Plant Molecular Biology, Department of Genetics, University of Tübingen, Germany

**Keywords:** Bipartition networks, large-scale maximum likelihood analyses, neighbor-nets, RAxML, ribosomal DNA, ITS sequence motifs

## Abstract

The multi-copy internal transcribed spacer (ITS) region of nuclear ribosomal DNA is widely used to infer phylogenetic relationships among closely related taxa. Here we use maximum likelihood (ML) and splits graph analyses to extract phylogenetic information from ~ 600 mostly cloned ITS sequences, representing 81 species and subspecies of *Acer*, and both species of its sister *Dipteronia*. Additional analyses compared sequence motifs in *Acer* and several hundred Anacardiaceae, Burseraceae, Meliaceae, Rutaceae, and Sapindaceae ITS sequences in GenBank. We also assessed the effects of using smaller data sets of consensus sequences with ambiguity coding (accounting for within-species variation) instead of the full (partly redundant) original sequences. Neighbor-nets and bipartition networks were used to visualize conflict among character state patterns. Species clusters observed in the trees and networks largely agree with morphology-based classifications; of de Jong’s (1994) 16 sections, nine are supported in neighbor-net and bipartition networks, and ten by sequence motifs and the ML tree; of his 19 series, 14 are supported in networks, motifs, and the ML tree. Most nodes had higher bootstrap support with matrices of 105 or 40 consensus sequences than with the original matrix. Within-taxon ITS divergence did not differ between diploid and polyploid *Acer*, and there was little evidence of differentiated parental ITS haplotypes, suggesting that concerted evolution in *Acer* acts rapidly.

## Introduction

Molecular phylogenetic studies of maples, *Acer* L. (Sapindaceae), have relied mainly on the internal transcribed spacer (ITS) region of nuclear ribosomal DNA (rDNA) (Cho et al. 1996; Ackerly and Donoghue, 1998; Suh et al. 2000; Pfosser et al. 2002; Tian et al. 2002). To the extent that they overlapped in species sampling, these studies have yielded contradictory relationships, although usually without statistical support. The ITS region used in these studies is part of the rDNA cistron coding for the 35S pre-RNA, which consists of 5′ ETS (external transcribed spacer), 18S, ITS1, 5.8S, ITS2, 25S, and 3′ ETS, with ITS1 having evolved from an intergenic spacer and ITS2 from an expansion segment in the rDNA large subunit (Hershkovitz and Zimmer, 1996; Hershkovitz et al. 1999). Plant genomes have thousands of copies (Hemleben et al. 1988) located in one or several loci, distributed on one or several chromosomes, and hybrid or allopolyploid individuals can contain different parental rDNA repeats (Volkov et al. 2004; Volkov et al. in press). Functional and pseudogenic copies can recombine, further increasing sequence variation within individuals. For all these reasons, signal contained in ITS sequences cannot always be mapped onto bifurcating trees, although concerted evolution constantly homogenizes new variation among the numerous rDNA repeats of an individual (Sanderson and Doyle, 1992; Suh et al. 1993; Baldwin et al. 1995; Wendel et al. 1995; Buckler et al. 1997; Volkov et al. 1999; Muir et al. 2001; Álvarez and Wendel, 2003; Razafimandimbison et al. 2004; Grimm et al. 2005; Okuyama et al. 2005; Ritz et al. 2005; Won and Renner, 2005). Direct sequencing of this region has therefore long been the accepted practice, and indeed the ITS sequences used in studies of *Acer* (Cho et al. 1996; Ackerly and Donoghue, 1998; Suh et al. 2000; Tian et al. 2002) were obtained from direct sequencing.

An initial survey that involved 160 cloned ITS sequences from 84 individuals of *Acer*, representing 45 species and 20 subspecies, revealed non-identical copies in most individuals (Grimm, 2003). This required sampling multiple accessions of as many species as possible. *Acer* includes many polyploids, and species are known to hybridize, although the frequency and extent of hybridization in nature appear to be limited (van Gelderen et al. 1994; Pfosser et al. 2002). The genus contains at least 124 species (van Gelderen et al. 1994) of which almost half occur in China. The closest relative of *Acer, Dipteronia*, with two species, also is endemic to China. As sampling increased to currently more than 600 clones, including clones from all known polyploid species, it became clear that species were characterized not by single nucleotide (nt) substitutions, but by sequence motifs. Sequence motifs or elements are series of linked substitutions and/or insertions and deletions (indels) within a given region (Hershkovitz and Lewis, 1996; Hershkovitz and Zimmer, 1996; Grimm, 2003; Volkov et al. 2003; Volkov et al. 2004; Denk and Grimm, 2005). ITS motifs in length-polymorphic regions (LPRs, comprising up to 50 nt) have been found to be phylogenetically informative (Grimm, 2003). For the present study, we greatly increased sampling density to test whether ITS motifs might clarify contradictory relationships found in the previous studies of *Acer* and whether they can be traced within Sapindaceae or other Sapindales (to which *Acer* belongs; Bremer et al. 1998; Thorne, 2000; Harrington et al. 2005).

Several approaches are available to analyze contradictory signal within and between phylogenetic data sets. We here employ phylogenetic networks as well as comparison of bootstrap values on branches obtained with different inference methods. Networks graphically represent signal from mutations in sequences, sets of trees, or inferred genetic distances. Competing signal may arise from the stochastic substitution process, poorly fitting evolutionary models, or the heuristic nature of many tree search algorithms. Or it may result from hybridization, lineage sorting, or lateral gene transfer. Whatever the causes of contradictory signals, a rapid method to visualize their extent is a splits-based graph. Splits graphs comprise median networks from sequences, consensus networks from trees, and split decomposition and neighbor-nets from genetic distances (Bandelt and Dress, 1992; Huson, 1998; Huber et al. 2002; Bryant and Moulton, 2002; Holland and Moulton, 2003; Bryant and Moulton, 2004; Holland et al. 2005; Kennedy et al. 2005; Morrison, 2005; Winkworth et al. 2005; Huson and Bryant, 2006). In splits graphs, each set of parallel edges represents a split or bipartition of the data into non-overlapping groups, with edge lengths being proportional to the number of mutations supporting a particular split. Tree-like parts of the diagrams represent un-contradicted patterns, whereas box-like structures represent conflict. Compared to a visual screening of alternative bifurcating trees, splits-based graphs allow a more thorough and qualitatively different assessment of alternative relationships supported by the data, especially if different types of splits graphs are employed.

Running full maximum likelihood (ML) analyses (e.g. computing 200 ML trees and 1,000 ML bootstrap replicates) under a parameter-rich model on matrices of some 600 sequences and 460 characters required a high-performance computational approach, such as implemented in RAxML (Stamatakis, 2006). RAxML-VI-HPC allows for ML-based computation of phylogenetic trees for datasets of up to 25,000 taxa with 1500 base pairs or 2,100 taxa with up to 50,000 base pairs (Stamatakis, 2006). We also used RAxML-based bootstrap analyses to explore the effects of using the several hundred original ITS sequences as opposed to using smaller matrices of consensus sequences that used ambiguity coding to account for within-species and subspecies variation.

Our multi-tiered approach to extracting phylogenetic signal from the multicopy ITS region thus combined (i) maximum likelihood tree inference from matrices of different composition, (ii) networks for exploring contradictory signal, and (iii) motif analyses at different hierarchical levels. This study also presents the most thorough attempt so far to identify major clades in *Acer*, the largest tree genus of the northern hemisphere besides *Quercus*.

## Material and Methods

### Taxon sampling

A list of the 83 species and subspecies of *Acer* and *Dipteronia* included in the analysis with their sources and gene bank accession numbers given in Appendix 1. Species were chosen to represent all sections and series of van Gelderen et al. (1994; Table 1). Where possible, species identifications were confirmed by comparison with herbarium specimens. Taxonomic resources (e.g. Flora Europaea 1998–2006; ITIS 2006; USDA, ARS, National Genetic Resources Program 2006) disagree markedly in recognized species and subspecies; we decided to follow in principal the most recent monograph of *Acer* (van Gelderen et al. 1994). A total of 606 ITS sequences (579 clones plus 22 clones with missing data for either ITS1 or ITS2 and 5 directly sequenced PCR products obtained by S.-F. Huang, Department of Biology, University of Missouri-St Louis, personal communication, 2003) was generated and uploaded to GenBank and the EMBL database.

Outgroup selection relied on Harrington et al. (2005), who demonstrated that *Acer* and *Dipteronia* together are sister to a clade comprising *Aesculus* (13–19 species in China, Europe, and North America), *Billia* (two species in the neotropics), and *Handeliodendron* (one species in China). All belong in Sapindaceae and hence Sapindales (Bremer et al. 1998; Thorne, 2000; Harrington et al. 2005). ITS sequences of *Aesculus, Handeliodendron*, and more distant Sapindaceae cannot unambiguously be aligned with *Acer* and *Dipteronia* to a sufficient degree. Hence, only *Dipteronia* could be included for alignment-based analyses. For motif analysis, we downloaded all ITS sequences of other families in the Sapindales from GenBank (December 2005): Anacardiaceae, 62 accessions, Burseraceae, 119 accessions, Rutaceae, 16 accessions, Meliaceae, 72 accessions, and Sapindaceae, 15 accessions. Sequences of *Handeliodendron* were generated for this study.

### DNA isolation, amplification, sequencing, and alignment

Isolation of total DNA followed a modified cethyltrimethylammonium bromide (CTAB) protocol (Gebhardt et al. 1989). DNA amplification by the polymerase chain reaction (PCR) was carried out with Vent®-polymerase (Roche Diagnostics GmbH, Mannheim, Germany) and the plant specific primers ITS-A and ITS-D (Denk et al. 2002; modified after Jobst et al. 1998), which amplify the 3′ end of 18S rDNA, ITS1, the 5.8S rDNA, ITS2, and the 5′ end of 25S rDNA. Amplified fragments were purified with QIAquick gel extraction kits (QIAGEN, Hilden, Germany). Ligation and transformation were performed using a pUC18 vector *E. coli* strain DH5α system. Up to 15 positive clones per sample were cultivated overnight, mixed 1:1 with glycerin, and stored at −70°C for plasmid isolation and data documentation. Cultures are available upon request. The DNA of up to ten clones per sample was isolated with High Pure Plasmid Isolation Kit® (Roche) and prepared for sequencing. Sequencing was done on an ABI Prism® automated sequencer with the universal primer M13 forward and reverse primers, and sequences were then edited and subsequently aligned with CHROMAS® V.1.45 (Technelysium Pty, Tewantin, Australia) and SeqMan II® plus MegAlign® (DNAStar, Madison, U.S.A.). Alignments were optimized manually, and for regions with length polymorphisms, we followed a protocol developed by Grimm (2003, p 11), taking into consideration results from independently computed alignments for each subspecies, species, or well supported clades to optimize the placements of gaps.

### Phylogenetic analyses

Parsimony analyses relied on PAUP version 4.0b.10 (Swofford, 2002), and Bayesian analyses on MrBayes version 3.1 (Huelsenbeck and Ronquist, 2001; Ronquist and Huelsenbeck, 2003). Maximum likelihood analyses used RAxML-VI-HPC (Stamatakis, 2006; software available at icwww.epfl.ch/~stamatak); with computations performed on the computer cluster of the Cyber-Infrastructure for Phylogenetic Research project (CIPRES, www.phylo.org) at the San Diego Supercomputer Center. Distance analyses were performed with neighbor-joining (NJ) and neighbor-net (NN) algorithms implemented in PAUP and SplitsTree version 4.3 (Huson and Bryant, 2006; available at www.splitstree.org).

One matrix consisted of 584 original ITS sequences. Reduced matrices were built as follows: (1) A matrix of 101 original sequences was used for computation of some NN splits graphs. (2) A matrix of 105 sequences included one consensus sequence per species or subspecies as long as these taxa exhibited minor (≤1 nt long) nucleotide polymorphism among ITS clones. Taxa that exhibited length polymorphisms or distinct ITS variants were represented by several consensus sequences. Arboretum samples that deviated from the wild type of the respective species were represented by additional consensus sequences. (3) A matrix of 40 sequences included one to three semi-strict (as defined below) consensus sequences for each of the species groups that had diagnostic ITS motifs high and (>75%) ML bootstrap support. To create semi-strict consensus sequences, intraspecific variability was ignored as long as it was restricted to a single species/subspecies. For consensus sequences that represented more than one species, mutational divergence was retained by using ambiguity coding.

Parsimony analyses were performed using the parsimony ratchet analysis for PAUP (PRAP) command block (Müller, 2004), with ten random taxon-addition replicates. Bayesian analyses used one cold and three incrementally heated Monte Carlo Markov chains (MCMC) in two simultaneous runs. Chains were run for 1 million cycles, with trees sampled every 100th generation, each using a random tree as a starting point and a temperature parameter value of 0.2 (the default in MrBayes). The first 298 trees of each run were discarded as burn-in; converging log-likelihoods, potential scale reduction factors for each parameter, and inspection of tabulated model parameters suggested that stationarity had been reached thereafter. The remaining trees were used to compute posterior probabilities of nodes.

Models for minimum evolution (distance) and Bayesian analyses were selected from the 24 models implemented in MrModeltest 2.1 (Nylander, 2004) employing the Akaike information criterion (AIC). Best model decisions from MrModeltest were compared to best models found for the same data via simultaneous evaluation of the 56 models implemented in DT-ModSel (Minin et al. 2003). The latter uses a Bayesian information criterion based on decision theory to gauge the different models’ performance in terms of branch-length error and degree of over-fitting. For the 105-taxon data set (84 species and subspecies), the general time-reversible (GTR) model plus a gamma shape parameter (Γ) and a proportion of invariable sites (I) received the best AIC score in MrModeltest, while the less parameter-rich transition model (TiM) plus Γ scored best in DT-ModSel. TiM + Γ cannot be specified in MrBayes, so we opted for the next parameter-rich model (GTR + Γ). Parameter estimation in MrBayes ran for the duration of specified MCMC runs. For the RAxML analyses, we also used the GTR + Γ model, with model parameters estimated over the duration of specified runs. Distance analyses (NJ) were performed under the GTR + Γ model and the HKY + Γ + I model (Hasegawa et al. 1985), using the parameter values found with MrModeltest.

Clade support was assessed with posterior probabilities (PP) computed with MrBayes and non-parametric bootstrapping (Felsenstein, 1985) as implemented in PAUP and RAxML. Bootstrap support (BS) under parsimony (BS_P_) and neighbor joining (BS_NJ_) is based on 10,000 replicates. Parsimony bootstrap replicates used a simple taxon addition tree as the starting point, tree-bisection-reconnection swapping, and one tree held in memory; more computation-intensive heuristic approaches have been shown not to increase the reliability of bootstrapping (Müller, 2005). Bootstrap support under ML (BS_ML_) is based on 1,000 replicates computed with the parallel message-passing-interface-based version of RAxML-VI-HPC on a LINUX cluster.

Alternative splits in bootstrap replicates and in the Bayesian partitions table were visualized as split networks with SplitsTree. In addition, we computed 200 ML trees on the original 584-taxon alignment in parallel, using 200 distinct randomized MP starting trees produced by RAxML. The advantage of using randomized MP starting trees is that the ML-based search starts from different points in the vast search space and it is thus less likely to get stuck in local ML maxima.

Neighbor-net (NN) splits graph analysis (Bryant and Moulton, 2002), implemented in SplitsTree, was used to infer the distribution of incompatible splits. Neighbor-net starts with genetic distances. In addition, bipartition networks were obtained by coding the partitions tables from RAxML, PAUP, and MrBayes as split matrices for SplitsTree. The frequency of each split becomes an edge length in the bipartition network, and contradictory splits can thereby be visualized. Splits graphs used either uncorrected p-distances or distances computed under HKY + Γ + I, with the model parameters found by MrModeltest (above).

Mutational patterns within length polymorphic regions (LPR1 and LPR2, see below) were treated as logically dependent (linked) characters and as one sequence motif. The ITS1 and ITS2 regions of *Acer* and *Dipteronia* were also screened for sequence elements conserved among major clades (conserved motifs). For motif analysis, variants were placed next to each other in a network that minimized the number of mutational changes between adjacent motifs. Motif variants were then mapped onto a constrained topology to visualize mutational trends and differentiation levels. The obtained motif differentiation pattern for *Acer* and *Dipteronia* was compared to homologous DNA stretches in other Sapindales.

## Results

### Phylogenetic relationships in *Acer*

We generated 606 ITS sequences from 231 leaf samples, representing 81 species and subspecies of *Acer* and both species of *Dipteronia*. A table summarizing sequence length, GC content, and sequence divergence for the amplified regions ITS1, 5.8S rDNA, and ITS2 is available from the first author. Data matrices have been uploaded to TreeBase (accessions SN2898–11606 to 11608; fully annotated NEXUS files for SplitsTree and PAUP/MrBayes can be obtained upon request). The 18S, 5.8S, and 25S rDNA gene portions were excluded from all phylogenetic analyses, as were ten ITS clones judged to be pseudogenes based on increased AT content and deletions within their 5.8S sequences (not shown). We also excluded two highly divergent and length polymorphic regions (LPRs), one of 49 bp in the ITS1 region and one of 53 bp in the ITS2 region (subsequently referred to as LPR1 and LPR2); these polymorphic regions were only included in some NN analyses (as specified below). In total, 584 ITS sequences (mostly cloned) were included as original taxonomic units.

**Figure 1:**
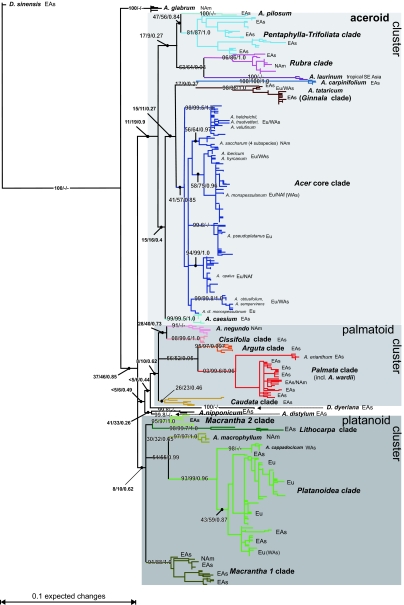
Phylogram representing the optimal bifurcating solution under ML computed with RAxML based on 584 ITS clones (including five directly sequenced PCR products). Bootstrap support (BS; in %) and posterior probabilities (PP) are shown for selected nodes as follows: BS from the 584 original sequences/BS from the 105 consensus sequences/PP from the 105 consensus sequences. Abbreviations: EAs, East Asia; Eu, Europe; NAf, North Africa; NAm, North America; WAs, Asia Minor.

**Figure 2:**
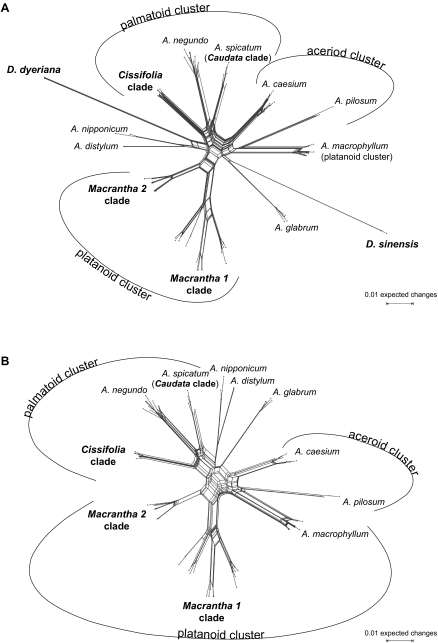
Neighbor-nets for *Acer* and *Dipteronia* computed with uncorrected p-distances. **A.** Neighbor-net based on 101 original sequences with the length-polymorphic regions LPR1 and LPR2 included. **B.** Neighbor-net for the same accessions, except that *D. sinensis* and *D. dyeriana* (six clones) were excluded.

**Figure 3:**
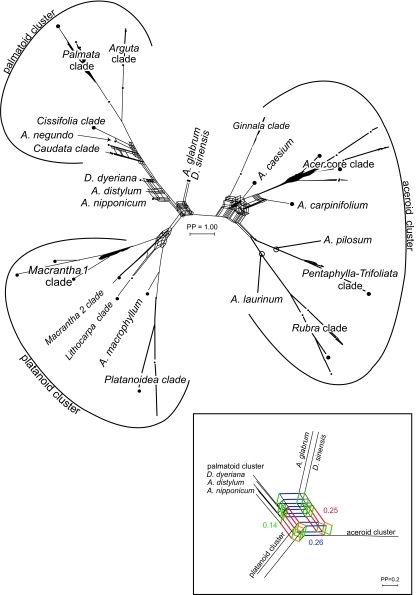
Bipartition network based on Bayes partitions for the 105-consensus-sequence matrix, representing 84 taxa of *Acer* and *Dipteronia,* based on the 19,406 post-burnin trees from two parallel MCMC runs. Edge lengths are proportional to the frequency of particular splits (bipartitions). The aceroid, platanoid, and palmatoid clusters each are supported by sets of alternative and semi-congruent splits (box-like structures). Inset: Enlarged central box portion of the splits graph, illustrating phylogenetic interference. The three most frequent alternative splits indicate that *A. glabrum* + *D. sinensis* are either sister to the aceroid cluster (blue), the palmatoid cluster, *D. dyeriana, A. nipponicum,* and *A. distylum* (red), or the platanoid cluster (green). Less frequent splits are semi-congruent to the most frequent splits. For example, the orange split (semi-congruent to the green split) groups *A. glabrum* + *D. sinensis* with *A. macrophyllum* and the *Platanoidea* clade.

All methods recovered three supraspecific groups: the aceroid, the palmatoid, and the platanoid cluster (Figs. 1–3), and many of the traditional sections and series of *Acer* (Table 1) were also recovered under ML (Fig. 1; TreeBase accession SN2898-11610), in the neighbor-net (Fig. 2), and in the bipartition network (Fig. 3). Traditional groups that have low statistical support (with all matrices and methods) were the *Acer* core clade and the *Caudata* clade (Fig. 1). Nevertheless, both were recovered in the best-known ML tree obtained from the 584 original sequences included in the largest matrix.

Molecularly well-supported groupings (Fig. 1) not previously recognized based on morphology were (i) the placement of *A. wardii* inside the *Palmata* clade, (ii) that of sections *Pentaphylla* and *Trifoliata*, and (iii) that of *A. campbellii* subsp. *campbellii* (sect *Palmata* ser *Sinensia* sensu de Jong, 1994) with *A. tschonoskii* (sect. *Macrantha*; the clade is labeled *Macrantha 2* in all our figures).

The geographic distribution of the major clades seen in the ITS data is shown in Fig. 1. Six clades of two or more species/subspecies are endemic in East Asia, four occur in East Asia and North America, two occur in Western Eurasia and East Asia, and one is widespread in the northern hemisphere.

The two species of *Dipteronia*, the sister genus to *Acer*, did not group together in the ML tree (Fig. 1) or any of the bipartition networks (Fig. 3 and Appendix 2). To investigate this unexpected result, we computed NN splits graphs from the 584-sequence matrix and from a 101-sequence matrix (of original sequences, not consensus sequences) and excluded/included the six clones representing *D. dyeriana* and *D. sinensis* as well as the LPR1 and LPR2 regions. In none of these experimental analyses did *D. dyeriana* and *D. sinensis* group together, although the previously seen aceroid, palmatoid and platanoid clusters were nearly always recovered (Table 2; Fig. 2, additional graphs not shown). The main effect of excluding the LPR1 and LPR2 regions was that species of the *Macrantha 2* clade separated from the platanoid cluster (Table 2). With genetically distant taxa excluded, the LPR1 and LPR2 could be aligned unambiguously (as explained above, these polymorphic regions were excluded from other analyses). Resultant NN splits graphs (Fig. 2) agreed with those from the 584 sequences: The inclusion of *Dipteronia* species mainly affected relationships among basal *Acer* lineages (compare length of central edges and position of non-clustered *Acer* species in Fig. 2A and Fig. 2B).

Method-dependent topological differences (evaluated for the 105-consensus-sequence matrix) were restricted to nodes that received moderate to low support (PP <0.95, BS <75%), and there were no model-dependent topological differences between GTR + Γ and HKY + Γ + I NJ trees (not shown). Topological differences among the 683 most parsimonious trees obtained from the 105-consensus-sequence matrix were restricted to nodes near the leaves, as was the case for the 200 ML trees inferred from the 584-original-sequence matrix without bootstrapping. Nodes with posterior probabilities of >0.95 (and BS >75%) were consistently recovered in all trees, irrespective of optimality criterion, while all nodes that varied with method had low or moderate probabilities. On the other hand, several nodes found in all parsimony and ML trees had low posterior probabilities and bootstrap support (see Node Support).

### Effects of using consensus sequences

To explore the effect of using consensus sequences that “masked” within-species and among clone variation via ambiguity coding, we compared bootstrap support (using 1,000 RAxML-computed replicates) obtained with the 584 original sequences, the 105 consensus sequences, and the 40 consensus sequences. Generally, corresponding nodes, i.e. nodes defining the same groups of taxa, had higher bootstrap support with the 105-consensus- than the 584-original-sequence matrix (see Appendix 3). The weakly supported *Acer* core clade had a PP of 0.85 and a BS_ML_ of 57% with the 105-consensus-sequence matrix, while with the 584-original sequence matrix it had a BS_ML_ of only 41%. Using the 40- instead of the 105-consensus-sequence matrix increased bootstrap support only slightly and support for a few terminal nodes actually decreased. An exception from these general trends was the sister taxon relationship between *A. distylum* and *A. nipponicum* (Fig. 1), which received low ML bootstrap support (41%) with the 584-original-sequence matrix, 33% with the 105-consensus-sequence matrix, and 37% with the 40-consensus-sequence matrix.

### Node support

Many backbone nodes of the preferred ML tree (Fig. 1) have PP < 0.95 and BS < 75% (Appendix 3). Such low support can reflect absence of phylogenetic signal or contradictory signal. The bipartition networks (one of which is shown as Fig. 3) show that backbone nodes in the ML tree (Fig. 1) generally correspond to the longest edges. Where placements have poor support, as is the case for *A. caesium, A. carpinifolium*, the *Acer* core clade, and the *Ginnala* clade, alternative splits are almost equally probable (Fig. 3), but basically there is too little signal in the data. Low support for the *Macrantha 2* clade, however, appears due to alternative bipartitions, some of which indicate a closer relationship of *Macrantha 2* taxa to the palmatoid cluster, *A. distylum, A. nipponicum*, and *D. dyeriana*, while others pull *Macrantha 2* taxa to the platanoid cluster (Figs. 2 and 3, Appendices 2 and 3). In the case of *A. negundo*, the low support for the placement of *A. negundo* as sister to the *Cissifolia* clade (Fig. 1) is mainly due to a single alternative split involving *A. caudatum* subsp. *ukurunduense* of the *Caudata* clade (Fig. 3 and Appendices 2 and 3).

### Motif analysis

**Figure 4:**
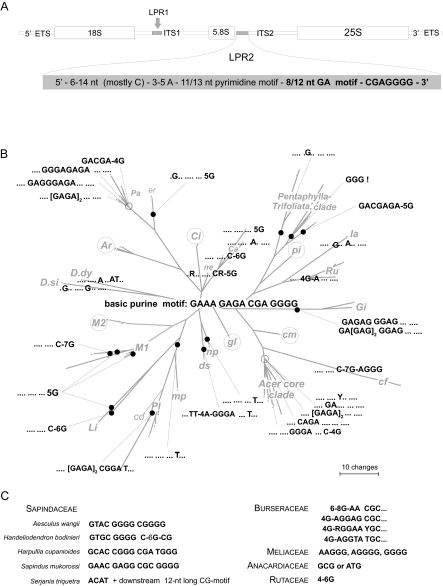
Evolution of the purine motif in the length polymorphic region LPR2. **A.** Diagram of the 35S rDNA cistron showing the position of LPR2 (5′ ITS2); the position of LPR1 (see text) is indicated by an arrow. **B.** Motif variants of the 15–20 nt long purine motif found in the LPR2 mapped onto an unrooted parsimony phylogram, with the monophyly of major clades enforced. Clades and species marked by open circles possess the unmodified basic LPR2 motif (center). **C.** Putative homologous variants of the LPR2 purine motif found in other Sapindaceae (left) and other families of the Sapindales (right). Abbreviations: Ar, *Arguta* clade; Ca, *Caudata* clade; Ci, *Cissifolia* clade; Gi, *Ginnala* clade; Li, *Lithocarpa* clade; M1, *Macrantha* 1 clade; M2, *Macrantha* 2 clade; Pa, *Palmata* clade; Pl, *Platanoidea* clade; Ru, *Rubra* clade; cd, *A. cappadocicum*; cf, *A. carpinifolium*; cm, *A. caesium*; ds, *A. distylum*; er, *A. erianthum*; gl, *A. glabrum*; la, *A. laurinum*; mp, *A. macrophyllum*; ne, *A. negundo*; np, *A. nipponicum*; pi, *A. pilosum; D.dy*, *D. dyeriana; D.si*, *D. sinensis*.

We screened all sequences for motifs, including the LPR1 and LPR2 regions (the location of which is shown in Fig. 4A). Examples of motifs found in *Acer-Dipteronia* and Sapindales are illustrated in Figures 4 (3′ part of LPR2) and 5 (3′ end of ITS1). Transitions are the most common substitutions, but appear restricted to specific motif positions.

The LPR1 of *Acer* and *Dipteronia* is 21 nt (clones of *A. buergerianum*) to 49 nt long (several *A. ibericum* clones) and is characterized by an initial motif of 6 to 7 G, followed by AG, a 20 nt long central motif, and an 8 nt long terminal pyrimidine motif. The initial GGGGGG-AG motif is conserved across the two genera. A generalized *Acer* LPR1 sequence is found in *A. caesium* (aceroid cluster), *A. negundo*, and all species of the *Arguta*, *Caudata* (except *A. caudatum* subsp. *ukurunduense*), and *Cissifolia* clades (palmatoid cluster), *A. macrophyllum*, all species of *Macrantha 2*, and all but one species of the *Platanoidea* clade (platanoid cluster). The remaining palmatoid and platanoid species, as well as *D. dyeriana* and *D. sinensis*, have LPR1 motifs that differ in 1 to 2 nt from the consensus sequence. Increased motif divergence, including prominent (≥3 nt long) length polymorphism, is restricted to the aceroid cluster. The LPR1 of *Acer* and *Dipteronia* cannot unambiguously be aligned with the remaining Sapindales. Sapindaceae other than *Acer* and *Dipteronia* all lack the initial GGGGGG-AG motif and the 8 nt long terminal pyrimidine motif. The 5′ end of the LPR1 central motif of Burseraceae (119 accessions, representing 7 genera) sometimes differs only in 1 nt from the 5′ end of the central motif of *Acer* and *Dipteronia*, and, as in *Acer* and *Dipteronia*, the LPR1 region of Anacardiaceae (62 ITS accessions, representing 12 genera), Burseraceae, and Rutaceae (16 accessions from 5 genera) is less than 30 nt long and has a 5′ G-dominated motif and a 3′ C-dominated motif. Unlike in Sapindaceae, however, major (≥3 nt long) intra- and intergeneric length polymorphism in these families is uncommon. In the Meliaceae (72 accessions from 10 genera) and *Murraya* (Rutaceae; 2 species sequenced), the LPR1 equivalents have increased length (55 to 65 nt) and point mutational variability.

The LPR2 (Fig. 4) of *Acer* and *Dipteronia* starts with a C-dominated motif (≤14 nt long) that is followed by three to five purines (predominantly 3 A) and a motif with pyrimidines (C and T), which is followed downstream by a purine-dominated motif (A and G; Fig. 4B). The 3′ end of LPR2 is defined by ACTTGGCC (with some taxon-specific modifications) downstream of the LPR2. Further LPR2 motifs characterize subspecies, species, or species groups: For example, (i) most species have an 11 to 20 nt long pyrimidine motif, [CCCCT]_2_CTC or CCCCT[CTC]_2_, usually accompanied by a 15 to 20 nt long purine motif that can be generalized as GAAA-[GAGA]_1–2_-CGA-GGGG (Fig. 4B) (ii) Other species differ in two to five point mutations and indels from the basic motif (shown in the center of Fig. 4B). (iii) In the *Pentaphylla-Trifoliata* clade (Figs. 1, 4B) and in several clones of *A. palmatum* subsp. *amoenum*, the downstream pyrimidine-purine motifs are partially deleted.

The LPR2 regions of *Dipteronia*, while differing from each other and from *Acer*, can still be aligned with each other, and LPR2-homologous DNA stretches are detectable in the remaining Sapindales. In Sapindaceae, they are 23 to 41 nt long and start with a C-dominated motif that ends with two or three purines (AA, GAA, AAG), followed by up to 15 downstream pyrimidines and a purine-dominated terminal part (Fig. 4C). In Burseraceae, the LPR2 starts with two C-dominated motifs (each with 6 or more Cs); Anacardiaceae and Meliaceae have three 5′ purines after the initial C-dominated motif, often three A, and then a 14 nt long motif comprising all four nucleotides in more or less equal frequencies; in Rutaceae (for which we screened 21 ITS2 accessions from five genera), the LPR2 is mainly composed of 5′ Cs and 3′ Gs (Fig. 4C).

**Figure 5:**
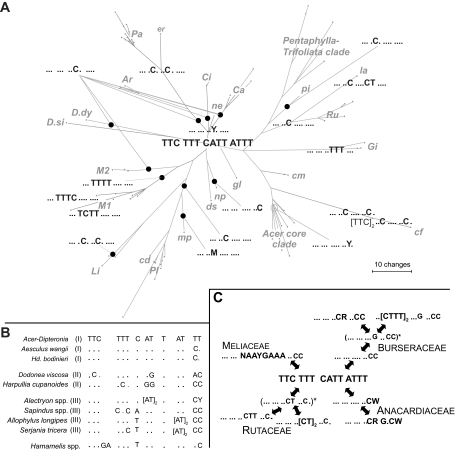
Evolution of a T-dominated ITS1 motif in 84 species and subspecies of *Acer* and *Dipteronia*, using the same parsimony framework as in Fig. 4. **A.** Within *Acer* lineages, single substitution events suffice to derive motif variants from one another. **B.** Evolution of the same motif among Sapindaceae genera. Motif variants in *Acer, Dipteronia, Aesculus wangii,* and *Handeliodendron (Hd.) bodinieri* are more similar to *Hamamelis,* a basal eudicot, than to other Sapindaceae. Roman numerals in parentheses refer to major Sapindales clades sensu Harrington et al. (2005). **C.** Evolution of the same motif among the Sapindales families Anacardiaceae, Burseraceae, Meliaceae, and Rutaceae. The same point mutations that account for motif variability within *Acer* are found among these families. Abbreviations as in Fig. 4.

In addition to the LPR1 and LPR2 ITS regions, we screened the remainder of ITS1 and ITS2 for clade-conserved motifs (approx. 12 to 20 nt long; Fig. 5 and Appendix 4). Motif variants of sister taxa usually involve the fixation of a single mutation (mostly transitions), and the detected variants can be ordered parsimoniously within species clusters. Examples are the permutations of the T-dominated ITS1 motif illustrated in Figure 5. Different clades of *Acer* have fixed substitutions of a C at specific positions in this motif. The last two nucleotides of the motif, TT or CT in *Acer* and *Dipteronia* and predominantly CT, TC, CC in other Sapindales, are relatively conserved. For example, 190 accessions of Burseraceae, representing seven genera, all had the CC ending.

More ‘ancestral’ and more ‘derived’ motif variants can co-exist within *Acer* individuals, subspecies, or species, or may be confined to taxon clusters, as indicated in Figure 5A.

The above-described motifs in the LPR1 and LPR2 regions plus eight motifs in the remainder of ITS1 and ITS2 (Appendix 4) support relationships found in the bipartitions network and the NN splits graphs (Figs. 2 and 3). Motifs also weakly support the monophyly of *Dipteronia*, but the two species have different mutational trends, with the ITS of *D. sinensis* exhibiting a bias towards C/G substitutions compared to *D. dyeriana* (Fig. 4B).

### Polyploidy and ITS divergence in *Acer*

To assess whether polyploid maples have increased ITS divergence, we compared as many diploid/polyploid pairs (with similar cloning efforts) as possible. Uncorrected p-distances in four pairs were:

- *A. monspessulanum* subsp. *monspessulanum* (*Acer* core clade, diploid), 56 clones from 14 individuals: 0–0.013; 38 clones from 10 individuals: 0–0.013; 12 clones from three individuals: 0–0.009; *A. opalus* (*Acer* core clade, diploid), 32 clones from 12 individuals: 0–0.009; *A. pseudoplatanus* (*Acer* core clade, tetraploid), 38 clones from 13 individuals: 0–0.015.
- *A. sempervirens* (*Acer* core clade, diploid), 19 clones from 4 individuals: 0–0.009; *A. velutinum* (*Acer* core clade, tetraploid), 19 clones from 3 individuals: 0–0.014.
- *A. rubrum* (*Rubra* clade, hexa- to octopolyploid), 9 clones from 3 individuals: 0–0.007; *A. saccharinum* (*Rubra* clade, tetraploid), 12 clones from 3 individuals: 0–0.013.
- *A. buergerianum* (*Pentaphylla*-*Trifoliata* clade, diploid): 4 clones from an arboretum: 0.003–0.008, 6 clones from the wild: 0–0.015; *A. griseum* (*Pentaphylla-Trifoliata* clade, diploid): 4 clones from an arboretum: 0.004–0.01, 4 clones from the wild: 0–0.005; *A. laurinum* (diploid), 4 clones from the wild: 0.001–0.007, *A. carpinifolium* (tetraploid), 4 clones from an arboretum: 0–0.008.

In *Acer ibericum* (erroneously synonymized under *A. monspessulanum* by Yaltirik, 1967), we found two co-existing ITS haplotypes in all four individuals (11 clones). Both variants share the *A. ibericum*-typical mutations and are undergoing concerted evolution. The ploidy level of *A. ibericum* is not known. Similar cases of co-existing haplotypes were discovered in *A. campestre* and *A. mono* subsp. *mono*.

## Discussion

The main goal of this study was to assess whether ITS data can be used for inferring the phylogeny of *Acer* in spite of intra-individual and intra-specific sequence divergence. Earlier phylogenetic studies of the *Acer* have all relied on directly sequenced ITS data (Cho et al. 1996; Ackerly and Donoghue, 1998; Suh et al. 2000; Tian et al. 2002), and obtained largely unsupported trees. Results obtained here with a ML analysis of 584 ITS sequences from multiple accessions of most species show that sequences largely group by species, putting to rest suspicions that ITS might be a dubious phylogenetic marker in *Acer*. We found no evidence of increased ITS divergence in wild material as opposed to trees from botanical gardens or in polyploid as compared to diploid species. Polyploidy can be accompanied by the coexistence of several nucleolus organization regions (NORs; Leitch and Bennett, 1997), and several NORs can also coexist in experimental hybrids (Komarova et al. 2004). Assuming that most polyploids are allopolyploids (Leitch and Bennett, 1997), one might have expected that coexisting NORs from different parents would result in sets of divergent ITS variants (homoeologs). Polyploid species of *Acer* are highly concentrated in the aceroid cluster, but there was no difference in within-species ITS divergence among polyploid and diploid species. We also found little evidence of within-individual co-occurrence of different parental ITS haplotypes. For *Nicotiana* it has been shown that in stabilized allopolyploids, one parental rDNA lineage can (but need not) be completely eliminated, while the other undergoes significant restructuring (Volkov et al. 1999; Lim et al. 2000; Volkov et al. 2004; Volkov et al. in press). The scarcity of divergent ITS variants in diploid and polyploid species of *Acer* cannot be fully understood without the investigation of experimental hybrids, but probably indicates rapidly acting concerted evolution.

While our results justify the use of ITS as a phylogenetic marker in *Acer* (Cho et al. 1996; Ackerly and Donoghue, 1998; Suh et al. 2000; Tian et al. 2002), they also show that intra-specific ITS divergence is sufficiently high to require inclusion of multiple sequences per species. Construction of consensus sequence matrices, however, is not obsolete because such matrices allow more complete searches as well as being visually more easily understood. Using 105 or 40 consensus sequences rather than the 584 original sequences resulted in slightly improved bootstrap support, probably because of slightly reduced contradictory signal and the more complete searches of a reduced tree space. The search space given by the number of all possible trees is 1.07 · 10^1338^-times larger for 584 taxa compared to 105 taxa and 4.55 · 10^1476^- times compared to 40 taxa. Reduction from 105 to 40 taxa reduces the number of possible trees by the factor 4.25 · 10^138^. Despite these huge differences in the size of the search space, matrix size had no significant effect on topology, indicating the efficiency of heuristic search algorithms.

The unsatisfactory (contradictory) results in some of the earlier ITS-based phylogenetic studies of *Acer* to some extent may be due to paralogy problems, but likely also to sparse taxon sampling and direct sequencing, resulting in numerous ambiguous base calls (a tabulation of GenBank *Acer* sequence quality is included in Grimm, 2003). Just as found here, *Dipteronia sinensis* in these earlier studies was nested inside *Acer*, and *D. dyeriana* and *D. sinensis* did not group together (Pfosser et al. 2002; Tian et al. 2002). Using multiple newly generated sequences for these species, we found that their ITS contains very little signal. Moreover, there are identical mutational fixation trends in *Acer* and one of the two species of *Dipteronia*. Chloroplast sequences from four loci (*trnL* region, *rpl16* intron, *psbA*-*trnH* spacer, *rbcL* gene) generated for 62 species of *Acer* plus five Sapindaceae outgroups, including both *Dipteronia* species, strongly support the mutual monophyly of *Acer* and *Dipteronia* (S.S. Renner, L. Beenken, G.W. Grimm, A. Kocyan, R.E. Ricklefs, unpublished data).

Based on the fossil record, the initial radiation of *Acer* took place at the end of the Cretaceous (>65 Ma; Wolfe and Tanai, 1987; Boulter et al. 1996; McClain and Manchester, 2001), and much of the phylogenetic signal in the ITS region seems to have been overwritten since then. Nevertheless and as noted in a benchmark study of seed plant ITS (Hershkovitz and Lewis, 1996), there are highly conserved sections, which correlate with phylogenetic divergence events as old as 350 Ma (also Hershkovitz and Zimmer, 1996). Analogously, basic ITS motifs could have survived in clades of *Acer* and other Sapindales since the Eocene.

A study of fossil *Acer* leaves and fruits from North America (Wolfe and Tanai, 1987) distinguished seven morphotypes from the Early and Middle Eocene (≥47Ma) and assigned them to extinct and extant sections. We sequenced several species from their extant sections, namely *A. caudatum* (two subspecies), *A. nipponicum*, *A. distylum*, and *A. spicatum*. Assuming that the fossils are correctly assigned, the earliest record of the *Distyla* lineage would be from the Late Eocene of East Asia (Tanai, 1983) and the earliest record of the *Spicata* lineage would come from the Middle Eocene of East Asia and the Late Eocene of Central Europe (Walther, 1972; Tanai, 1983). *Acer nipponicum* and *A. caudatum* belong to the geologically younger section *Parviflora* (Late Oligocene/Early Miocene, N America; Wolfe and Tanai, 1987). These species all place relatively basal in the ML tree or near the centers of networks (Figs. 1, 2, 3), and their sequence motifs are close to the inferred basic *Acer* ITS motifs (Fig. 4B and Appendix 4). In a broad sense, then, early appearance in the fossil record correlates with possession of basic ITS sequence motifs and relatively early divergence in the phylogenetic tree. More critical placements of fossils, however, are necessary to test this further.

The species of *Acer* have been grouped into many subgenera, sections, and series (Pax, 1885; Pax, 1886; Pojárkova, 1933; Momotani, 1962; Ogata, 1967; de Jong, 1976; Delendick, 1981; Delendick, 1982; Mai, 1984; de Jong, 1994; our Table 1). While these authors’ higher-ranked groups are not recovered by ITS data, ten of de Jong’s 16 sections are supported, either in the trees and networks, or in the cases of sections *Negundo* and *Macrantha* by characteristic motifs. Of de Jong’s 19 series, 14 are supported by trees, networks, or motifs. In addition, six species that have been difficult to place based on morphology are clearly assigned by the ITS data: *Acer carpinifolium* is closest to *A. caesium*, *A. tataricum* (Ginnala clade), and to species of the *Acer* core clade; *A. laurinum* is sister to the *Rubra* clade; *A. mandshuricum* and *A. pentaphyllum* belong in the *Pentaphylla-Trifoliata* clade; *A. pilosum* is nested within the aceroid cluster, where it is basal to either *A. laurinum* + *Rubra* or the *Pentaphylla-Trifoliata* clade; and *A. wardii* belongs to the *Palmata* clade (Fig. 1).

The results of this study show that the ITS region in *Acer*, when analyzed such that within-species variation is taken into account, contains clear phylogenetic signal, and the geographic clustering and overall agreement with morphologically defined taxa (species, series, sections) suggest that the relationships seen indeed mirror the evolutionary unfolding of maples.
